# Emotion potentiates response activation and inhibition in masked priming

**DOI:** 10.3389/fnint.2012.00109

**Published:** 2012-11-16

**Authors:** Bruno R. Bocanegra, René Zeelenberg

**Affiliations:** Department of Psychology, Erasmus University RotterdamRotterdam, Netherlands

**Keywords:** emotion, masking, motor priming, fearful faces, activation, inhibition

## Abstract

Previous studies have shown that emotion can have 2-fold effects on perception. At the object-level, emotional stimuli benefit from a stimulus-specific boost in visual attention at the relative expense of competing stimuli. At the visual feature-level, recent findings indicate that emotion may inhibit the processing of small visual details and facilitate the processing of coarse visual features. In the present study, we investigated whether emotion can boost the activation and inhibition of automatic motor responses that are generated prior to overt perception. To investigate this, we tested whether an emotional cue affects covert motor responses in a masked priming task. We used a masked priming paradigm in which participants responded to target arrows that were preceded by invisible congruent or incongruent prime arrows. In the standard paradigm, participants react faster, and commit fewer errors responding to the directionality of target arrows, when they are preceded by congruent vs. incongruent masked prime arrows (positive congruency effect, PCE). However, as prime-target SOAs increase, this effect reverses (negative congruency effect, NCE). These findings have been explained as evidence for an initial activation and a subsequent inhibition of a partial response elicited by the masked prime arrow. Our results show that the presentation of fearful face cues, compared to neutral face cues, increased the size of both the PCE and NCE, despite the fact that the primes were invisible. This is the first demonstration that emotion prepares an individual's visuomotor system for automatic activation and inhibition of motor responses in the absence of visual awareness.

## Emotion potentiates response activation and inhibition in masked priming

Previous studies have shown that emotion can have 2-fold effects on visual perception. At the visual object level, emotional stimuli benefit from a stimulus-specific boost in the allocation of visual attention which occurs at the relative expense of spatially or temporally competing stimuli (Fox et al., 2001; Bocanegra and Zeelenberg, 2009a). At the visual feature level, recent findings indicate that emotion facilitates the fast processing of coarse visual features (Phelps et al., 2006; Bocanegra and Zeelenberg, 2009b, 2011) and inhibits the slower processing of small visual details (Bocanegra and Zeelenberg, 2009b, 2011). In the present study, we investigated whether emotion influences covert visuomotor processing that occurs in the absence of overt visual perception.

Many authors have suggested that the primary function of affective reactions is to enhance an organism's preparedness for action (Dolan, 2002; Phelps and LeDoux, 2005; Hajcak et al., 2007; Yiend, 2010; Bradley et al., 2012). Indeed, it has been shown that the perception of fearful faces enhances corticospinal motor tract excitability (Schutter et al., 2008). Consistent with these ideas, emotion influences response times (RTs) in various experimental paradigms, such as visual search and cueing tasks. For example, many studies indicate that a task-irrelevant emotional cue can influence RTs to a visual feature of a subsequent target (such as, location, color, shape, or orientation) (Mogg and Bradley, 1999; Fox et al., 2001; Yiend and Mathews, 2001; Mathews et al., 2003).

It has been proposed that, emotion influences RTs to visual stimuli either by speeding up access to visual awareness (e.g., by engaging attention to a stimulus or a certain stimulus feature) or by enhancing the processes responsible for maintaining visual awareness (e.g., by sustaining attention to a stimulus or stimulus feature). Within most theoretical frameworks, emotional cues are thought to modulate the perceptual processing stages that result in the overt identification of a feature, which in turn influences the downstream activation of motor codes and subsequent response execution (for an overview, see Yiend, 2010, for a direct emotion modulation in motor processing, see Schutter et al., 2008; Bradley et al., 2012). If visual stimuli access or occupy overt stages of perception more readily, this, in turn, could accelerate or increase the build-up of response activation triggered by a response-contingent stimulus feature.

A modulation in visual perception provides a natural explanation of how emotion might influence RTs to a stimulus. As a general rule one can say that as the perceptual strength of a visual feature is increased or decreased, detection or identification times also decrease or increase respectively (Teichner and Krebs, 1974). However, several studies, which were not concerned with the impact of emotion on action, now indicate that the initial stages of motor responding do not depend causally on the conscious perception of the stimulus feature specifying the response to be executed. Instead, it is well-established that simple stimulus features (such as color, shape, or orientation) can trigger motor responses prior to visual identification (Klotz and Neumann, 1999; Eimer and Schlaghecken, 2003; Sumner et al., 2006). These covert visuomotor responses have generated much interest because they demonstrate that sensory information can trigger motor responses directly, by-passing the perceptual mechanisms that support conscious visual perception.

**Figure 1:**
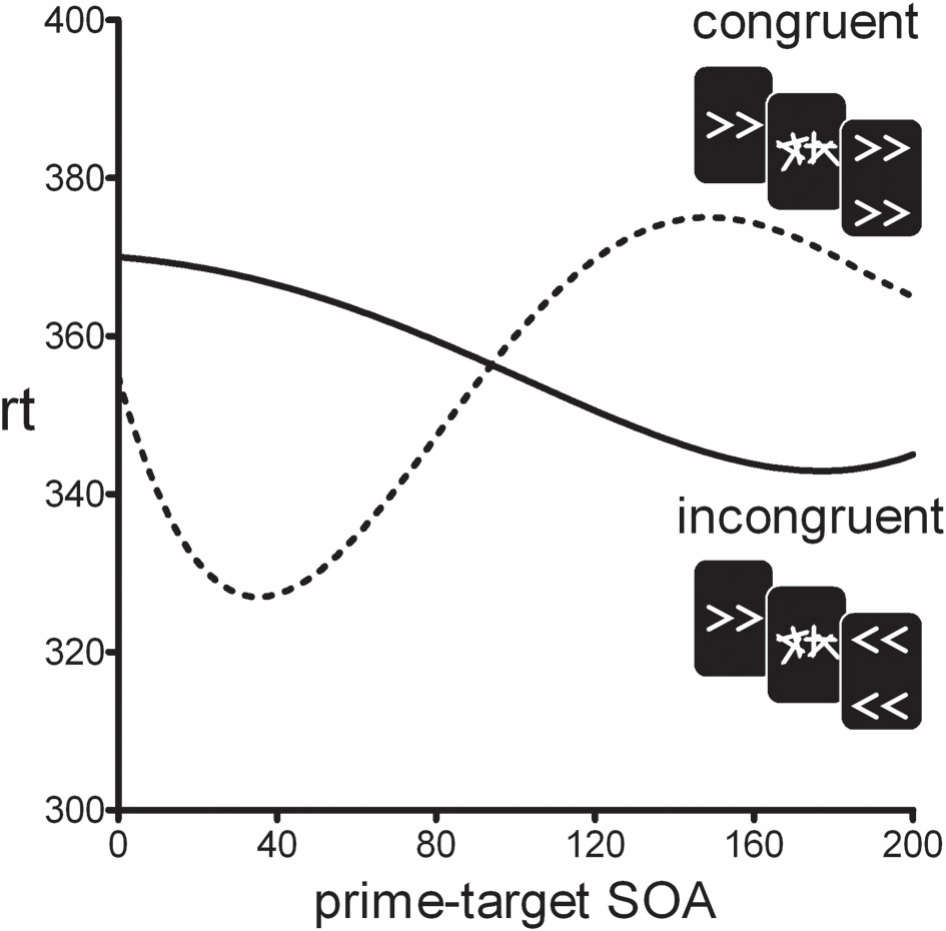
**Typical time-course of the positive congruency effect (PCE ≈0–100 SOA) and the negative congruency effect (NCE ≈100–250 SOA)**.

In the standard motor priming paradigm, a prime stimulus is backward-masked and followed by a clearly visible target stimulus. On congruent trials, prime and target are mapped on the same response, whereas on incongruent trials they are mapped on different responses. Although participants are unable to visually identify the masked primes, it has been shown that reaction-times differ for congruent vs. incongruent trials (e.g., Klotz and Neumann, 1999). Typically, prime and target are presented in rapid succession and positive congruency effects (PCEs) are observed (i.e., congruent trials are faster than incongruent trials). Surprisingly, however, when the prime-target stimulus onset asynchrony (SOA) is increased beyond 100 ms, the PCE turns into a negative congruency effect (NCE) where congruent trials are *slower* than incongruent trials (see Figure 1) (Eimer and Schlaghecken, 2003; Sumner et al., 2006). The initial PCE and subsequent NCE have been interpreted as an activation-followed-by-inhibition sequence reflecting the workings of low-level motor control mechanisms (Schlaghecken et al., 2006). Initially, the prime-induced response activation facilitates responding to the target on congruent trials, compared to incongruent trials. However, when the mask suddenly removes the sensory signal supporting the prime response, this specific motor response is actively inhibited which leaves the opposite response relatively more active. If a target is presented during this inhibition phase incongruent trials will be facilitated compared to congruent trials. This inhibitory mechanism has been interpreted as an “emergency brake” mechanism in covert visuomotor processing (Schlaghecken et al., 2006).

In light of these findings, an intriguing unexplored question is whether emotion modulates covert visuomotor processing *prior* to the overt perception of a visual stimulus. Conceivably, emotion might influence RTs in two distinct ways. On the one hand, emotion might modulate the perceptual strength of the conscious percept of a visual feature (Phelps et al., 2006; Bocanegra and Zeelenberg, 2011), and as a result of this, influence the build-up of motor activation that is initiated once the response-contingent stimulus feature is overtly identified. On the other hand, emotion might also influence covert visuomotor responses that are initiated prior to the identification of the initiating visual feature, which could influence RTs-independent of conscious visual perception (Vorberg et al., 2003). A crucial difference between these possibilities is that an overt perceptual mechanism predicts that RT-effects should depend critically on the response-contingent stimulus feature having been identified faster or more accurately (see Phelps et al., 2006; Bocanegra and Zeelenberg, 2009b), whereas covert visuomotor mechanisms predicts that RT-effects should be obtained even if the response-contingent feature of the stimulus has not been processed fully enough to be visually identified. Previous emotional studies did not address this distinction because in these paradigms motor responses were always elicited by a clearly visible suprathreshold stimulus (Yiend, 2010) or were elicited by a non-visual TMS pulse applied directly over the motor cortex (Schutter et al., 2008). Here, we employed a novel emotional cueing paradigm where a subthreshold prime is rendered invisible through pattern-masking in order to tap into the early activation phase and subsequent inhibition phase of covert visuomotor processing.

## Experiment 1

**Figure 2:**
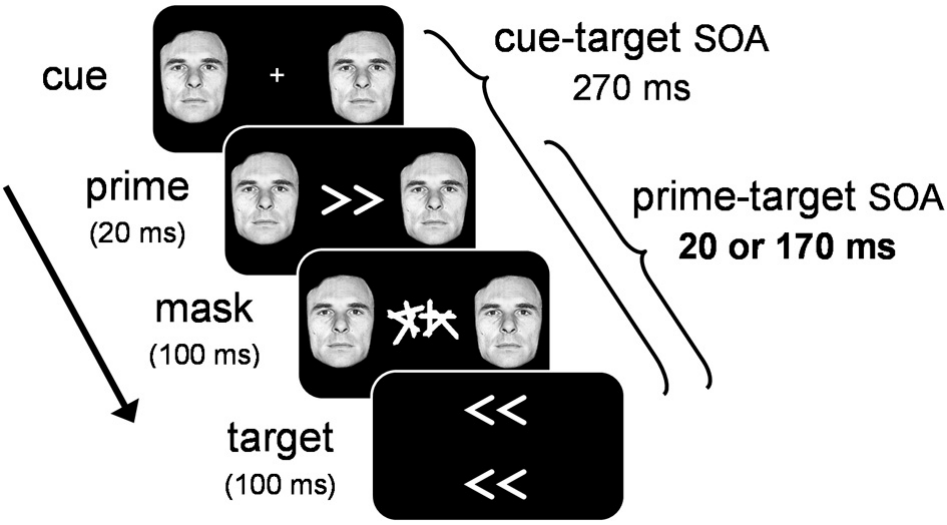
**Illustration of the general trial sequence for Experiments 1–3.** Target arrows were not presented in Experiment 3. See text for details.

In order to tap into covert visuomotor processing and minimize the effect of overt visual perception on RT, we used a masked priming paradigm where participants performed speeded responses to target arrows that were preceded by masked prime arrows (see Figure 2). On any given trial, the masked prime elicited either the same response as the target (congruent trials), or a different response (incongruent trials). By comparing the RT-differences between congruent and incongruent trials we assessed the covert visuomotor processing triggered by the masked prime through its effect on the subsequent target. Specifically, we assessed the PCE at a short prime-target SOA (20 ms) and the NCE at a long prime-target SOA (170 ms). To test whether emotion modulates covert visuomotor processing, we presented an emotional face cue concurrently with the prime and assessed the magnitude of the PCE and NCE. If emotion modulates covert visuomotor processing, larger prime-target congruency effects are expected when the prime is accompanied by an emotional face cue, compared to a neutral face cue.

## Methods

### Participants

Twenty undergraduate students at the Erasmus University Rotterdam participated for course credit or a small monetary reward. All were naïve as to the purpose of the study, reported normal or corrected-to-normal vision, and gave informed consent.

### Stimulus materials, apparatus, and procedure

Stimuli were presented on a gamma-corrected Iiyama 21-in. (100 Hz refresh-rate; 1600 × 1200 pixel resolution). A white fixation cross (0.8° × 0.8°) was presented at the center of a uniform black background for 500 ms prior to the stimulus sequence (see Figure 1). To manipulate emotion, we selected 11 *fearful* and *neutral* facial expressions from the Picture of Facial Affect (Ekman and Friesen, 1976). Cue displays consisted of a bilateral pair of fearful or neutral facial cut-outs of the same person (6.5° in diameter), presented left and right of fixation at 8° eccentricity. We chose to manipulate emotional significance with fearful facial expressions because previous studies have shown that this expression reliably activates the amygdala (Whalen et al., 1998), and has been shown to modulate perceptual processing throughout the visual system (Vuilleumier, 2005). Primes consisted of white left-pointing or right-pointing double arrows (<< and >>; size 3.5° × 1.8°) presented at fixation for 20 ms. A mask was presented for 100 ms immediately following the prime. Masks consisted of two white characters covering the entire area where primes had been presented. Targets consisted of two left-pointing or right-pointing arrow-pairs (3.5° × 1.8°), presented at 1.5° eccentricity above and below the center of the mask. The cue-target SOA was held constant (270 ms), and the prime-target SOA was either short (20 ms) or long (170 ms). Please note that by equalizing the cue-target SOA across the two prime-target SOA conditions, we created an inherent methodological confound between prime-target SOA and cue-prime SOA. Ten different mask characters were constructed, each consisting of four randomly oriented lines. To minimize prime-mask feature-overlap (Lleras and Enns, 2004), none of the lines in the mask shared the angular orientation of the prime. For each of the two mask positions, a character was sampled randomly from the set of ten mask characters.

Participants viewed the display at a distance of approximately 60 cm, maintaining central eye fixation, responding as quickly and accurately as possible to the direction of the target by pressing the “*z*” key for left and the “*m*” key for right. The experiment consisted of two blocks, one for the short SOA (20 ms) and one for the long SOA (170 ms). Each block was divided up into 5 experimental sub-blocks of 88 trials each. All experimental conditions within each sub-block were equiprobable and were presented in a randomized order. The order of the blocks was counterbalanced across participants.

### Data analysis

Incorrect responses were excluded from the analysis (<5% in all conditions). Mean RTs were calculated for correct responses, removing trials with RTs of less than 200 ms or more than 800 ms (1.6% of all trials). The same outlier criterion was used in all experiments reported here. A repeated-measures analyses-of-variance was conducted that included the factors prime-target SOA (20 ms vs. 170 ms), cue-type (fearful vs. neutral) and prime-target congruency (congruent vs. incongruent).

## Results and discussion

**Figure 3:**
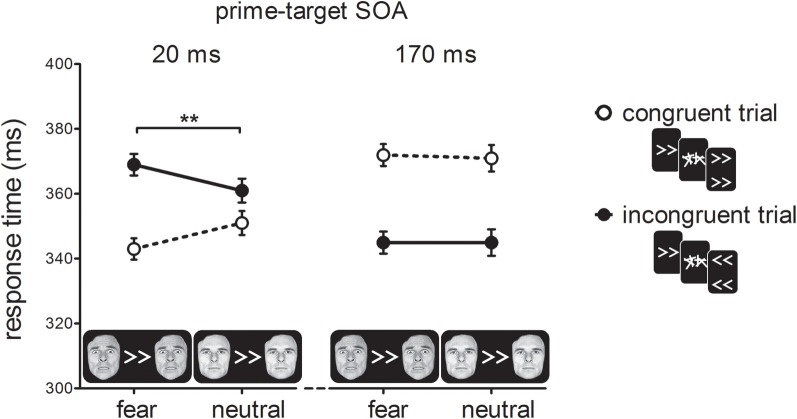
**Response times in milliseconds (ms) for the experimental conditions in Experiment 1.** For compatible trials, prime, and target arrows elicited the same response, whereas for incompatible trials they elicited different responses. Error-bars indicate within-subject standard errors of the mean difference (Loftus and Masson, 1994). ^**^p < 0.01 (interaction).

Figure 3 shows RTs as a function of prime-target SOA, cue-type and prime-target congruency. As expected, we found a cross-over interaction effect between prime-target congruency and SOA, *F*_(1, 19)_ = 100.93, *p* < 0.001, η^2^_*p*_ = 0.84. At the short prime-target SOA, we observed a PCE, *t*_(19)_ = 5.92, *p* < 0.001; congruent trials were 18 ms faster than incongruent trials. At the long prime-target SOA, we observed a NCE, *t*_(19)_ = 7.89, *p* < 0.001; congruent trials were 26 ms slower than incongruent trials.

Importantly, we observed a two-way interaction between cue-type and prime-target congruency, *F*_(1, 19)_ = 8.51, *p* < 0.001, η^2^_*p*_ = 0.31, indicating that the covert effect of the prime stimulus on target responding depended on the emotional significance of the cue stimulus. In addition, we obtained a three-way interaction between prime-target SOA, cue-type and prime-target congruency, *F*_(1, 19)_ = 20.36, *p* < 0.001, η^2^_*p*_ = 0.52. Cue-type and prime-target congruency interacted at the short SOA, *F*_(1, 19) = 27.00_, *p* < 0.001, η^2^_*p*_ = 0.59. Specifically, this shows that the PCE was larger for fearful cues (27 ms; *t*_(19)_ = 8.15, *p* < 0.001) than for neutral cues (10 ms; *t*_(19)_ = 2.68, *p* = 0.015). The NCE, however, was not affected by the emotional status of the face cues, as indicated by the lack of a cue-type × prime-target congruency interaction at a long SOA, *F* < 1, *p* > 0.75. Thus, the main finding of Experiment 1 was that the presentation of a bilateral fearful face cue potentiated the activation of the visuomotor response elicited by the masked prime.

## Experiment 2

Why was the PCE enhanced by emotion whereas the NCE was not? It has recently been proposed that the NCE may partly depend on global inhibitory processes that occur *between* response-channels (Praamstra and Seiss, 2005; Schlaghecken et al., 2006). In Experiment 1, the presentation of a bilateral fearful cue may have resulted in the same inhibitory balance between the response-channels as a bilateral neutral cue. If the NCE depends partly on the relative balance of lateral inhibition between the left vs. right response-channels, the symmetrical bilateral cues we used in Experiment 1 may not have been optimal in order to influence reciprocal inhibition between response-channels.

In order to test the possibility that emotion also enhances the NCE we presented the emotional stimulus unilaterally in order to influence the inhibitory balance between the response-channels (Praamstra and Seiss, 2005). In our second experiment, we did this by constructing cues consisting of a fearful face paired with a neutral face and varied their location (see Mogg and Bradley, 1999; Yiend and Mathews, 2001). Critically, if emotion enhances covert visuomotor processing, priming effects should be larger when the fearful face is presented in the hemifield that matches the primed response, compared to when the fearful face is presented in the hemifield that mismatches the primed response.

## Methods

Twenty students participated in the experiment. All experimental aspects were identical to Experiment 1 except for the type of cue displays used. Cue displays consisted of a fearful face paired with a neutral face that were presented left and right of fixation. On half of the trials the fearful face was presented left of fixation and the neutral face was presented right of fixation. On the other half of the trials the location of fearful and neutral faces was reversed. This resulted in two cue-types: cues where the location of the fearful face was congruent with the direction of the prime response (congruent fear cue), and cues where the location of the fearful was incongruent with the direction of the prime response (incongruent fear cue). Incorrect responses were again excluded (<6% in all conditions), and RTs were trimmed (1.6% of all trials).

## Results and discussion

**Figure 4:**
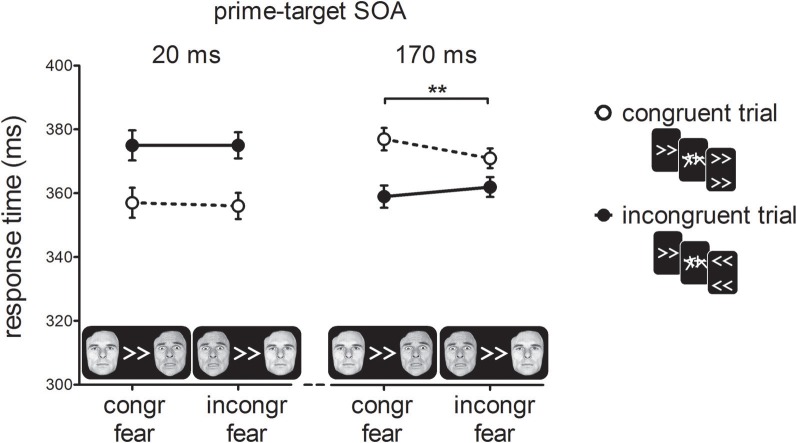
**Response times in milliseconds (ms) for the experimental conditions in Experiment 2.** For congruent fear cues, the fearful face was presented on the same side as the direction of the prime, whereas for incongruent fear cues the fearful face appeared on the opposite side. Error-bars indicate within-subject standard errors of the mean difference (Loftus and Masson, 1994). ^**^p < 0.01 (interaction).

Figure 4 shows RTs as a function of prime-target SOA, cue-type and prime-target congruency. As in Experiment 1, and consistent with previous reports (e.g., Eimer and Schlaghecken, 2003), we found a cross-over interaction effect between prime-target congruency and SOA, *F*_(1, 19)_ = 32.37, *p* < 0.001, η^2^_*p*_ = 0.63. At the short prime-target SOA, we observed a PCE, *t*_(19)_ = 4.53, *p* < 0.001; congruent trials were 18 ms faster than incongruent trials. At the long prime-target SOA, we observed a NCE, *t*_(19)_ = 4.54, *p* < 0.001; congruent trials were 13 ms slower than incongruent trials.

Importantly, we observed a two-way interaction between cue-type and prime-target congruency, *F*_(1, 19)_ = 7.95, *p* =0.01, η_p^2^ = 0.30_, indicating that the covert effect of the prime stimulus on target responding depended on the emotional significance of the cue stimulus. Although the three-way interaction between prime-target SOA, cue-type and prime-target congruency failed to reach significance, *F*_(1, 19)_ = 2.15, *p* = 0.16, η^2^_*p*_ = 0.10, we tested the two-way interactions between cue-type and prime-target congruency separately for the two SOA conditions to determine which of the SOAs was driving the two-way interaction between cue-type and prime-target congruency. Cue-type and prime-target congruency did not interact at the short SOA, *F*<1, *p* > 0.50, but did at the long SOA, *F*_(1, 19)_ = 11.42, *p* < 0.01, η^2^_*p*_ = 0.38, suggesting that the NCE was larger for fearful cues (18 ms; *t*_(19)_ = 5.21, *p* < 0.001) than for neutral cues (9 ms; *t*_(19)_ = 2.79, *p* = 0.01). Thus, the covert visuomotor inhibition of the motor response elicited by the masked prime was larger when the peripheral fearful face was presented in the hemifield that matched the response-channel activated by the prime.

## Experiment 3

Our findings in Experiments 1 and 2 suggest that emotion enhances the activation and inhibition of covert visuomotor responses elicited by an invisible masked stimulus. In Experiment 1, the presentation of a fearful face cue enhanced covert response activation and in Experiment 2 the peripheral presentation of a fearful face potentiated covert response inhibition when it was presented in the hemifield that matched the response-channel activated by the prime. Although none of the participants in Experiments 1 and 2 reported having seen any of the prime arrows, the different cue-types might have differentially affected any residual prime visibility. Conceivably, this could have influenced the magnitudes of the PCE and NCE (see Sumner et al., 2006). In order to address this possibility, we assessed prime identification performance for the cue displays used in Experiments 1 and 2.

## Methods

Ten additional students participated in the experiment. Participants performed a non-speeded prime identification task, indicating the direction of the primes by pressing “*z*” for left and “*m*” for right. It has been shown that the presence of a trailing target during a prime identification task makes it virtually impossible for participants to follow task instructions (Eimer and Schlaghecken, 2003). Thus, we excluded the target in order to prevent artificially reduced performance levels. Also, we included a short response delay (2 s) after mask presentation in order to ensure that identification performance would not be contaminated by any short-lived covert responses elicited by the masked primes (see Klapp and Hinkley, 2002). All other experimental aspects were identical to the previous experiments. Observers performed 2 blocks of 176 trials, one block containing the fearful and neutral cues, and the other containing the match and mismatch cues. Although the number of trials and participants are less than those included in Experiments 1 and 2, these numbers are comparable to other control experiments that have been published in the motor priming literature (Naccache and Dehaene, 2001; Klapp and Hinkley, 2002; Kiesel et al., 2007). The order of the blocks was counterbalanced across participants.

## Results and discussion

**Figure 5:**
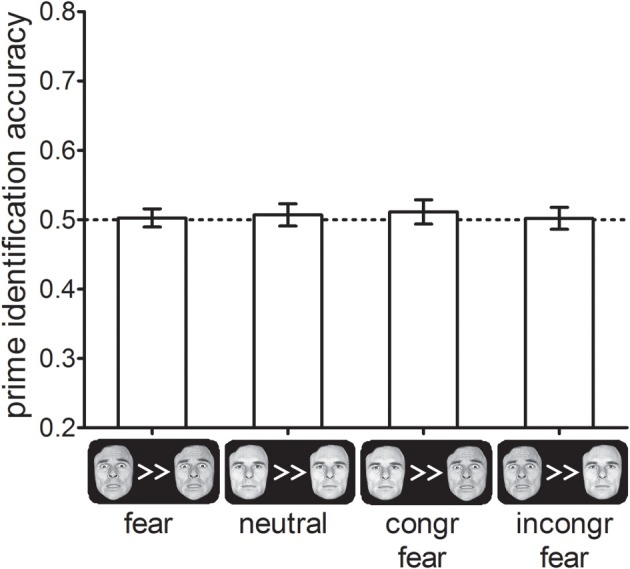
**Percentage correctly identified primes as a function of cue-types in Experiment 3.** Error-bars indicate standard errors of the mean.

Prime identification accuracy did not differ between the four different cue-types, *F*< 1, *p* > 0.8 (see Figure 5). In addition, identification accuracy did not differ significantly from chance performance (50%) in any of the cue-type conditions (all *t*s < 0.9, *p*s > 0.4), In addition, we calculated the JZS Bayes factor for all four conditions. Bayes factors can be used to provide confirmative evidence for the null-hypothesis of no effect by estimating how much more likely the null-hypothesis is given the data relative to the alternative hypothesis (Rouder et al., 2009). We found that for all cue-type conditions *JZS-BF*s >3, which is typically considered positive evidence in favor of the null-hypothesis by researchers advocating the use of Bayesian statistics. This suggests that the results of Experiments 1 and 2 were not mediated by the effect of the cues on prime visibility.

## General discussion

In the present study, we investigated whether emotion potentiates visuomotor responses that are initiated *prior* to the conscious visual identification of a stimulus. Specifically, we assessed the initial activation phase and subsequent inhibition phase of motor responses elicited by an invisible masked stimulus. In accordance with previously reported findings, we obtained a positive congruency effect (PCE) at a short (20 ms) SOA and a negative congruency effect (NCE) at a long (170 ms) SOA (for an overview, see Eimer and Schlaghecken, 2003). More important, in Experiment 1, the presentation of a bilateral fearful face cue, compared to a bilateral neutral face cue, increased the size of the PCE. The size of the NCE, however, was not affected by the emotional status of the face cue. In Experiment 2, we presented cues consisting of both a neutral face and a fearful face, and varied the location of the neutral and fearful face (e.g., left face: neutral, right face: fearful). The presentation of a fearful face cue at the location of the primed response, compared to a fearful face cue at the opposite location, increased the size of the NCE. Combined, these findings suggest that the presentation of fearful face cues potentiate both early facilitatory and later inhibitory stages of covert visuomotor processing, Experiment 3 indicated that prime visibility was at chance performance for each of the different cue-types used in Experiments 1 and 2, which suggests that our findings were not mediated by an effect of emotional cueing on prime visibility.

A reviewer pointed out to us that the lack of an emotional modulation of the NCE in Experiment 1 may have been due to an intrinsic experimental confound. In our stimulus sequence, we equalized the cue-target SOA for the short and long prime-target SOA conditions. In doing so, we inherently created an experimental confound between the prime-target SOA and the cue-prime SOA: the cue-prime was always shorter in the long prime-target SOA condition, compared to the short prime-target SOA condition. Thus, if the emotional modulation due to the bilateral fearful cues requires some time to build-up, the prime presentation in the long prime-target condition may have been too soon after cue-onset to be modulated by emotion. It is thus possible that with bilateral cues the NCE would also be modulated by emotional face cues if a longer cue-prime SOA is used. However, these considerations do not invalidate our primary conclusion that emotion modulates visuomotor processing prior to visual awareness.

Interestingly, a previous study has demonstrated that a masked subthreshold emotional cue presented outside visual awareness can nonetheless influence RTs to a subsequent target (Mogg and Bradley, 1999). A pair of emotional and neutral face stimuli were briefly displayed and masked in a dot-probe task. RTs were faster when the spatial location of the emotional face matched the location of the subsequent target compared to when it mismatched. This finding shows that emotional facial expressions may rapidly engage and release attention (see also Santesso et al., 2008; Maratos, 2011), and suggests that these attentional effects of emotion may also operate prior to overt visual awareness. Indeed, our results in Experiment 2 may have due to covert spatial attentional mechanisms. Although the (Mogg and Bradley, 1999) study relates to our finding in the sense that emotion may influence the allocation of attention prior to visual awareness, there is a critical difference with our study. In the Mogg and Bradley (1999) study, the task-irrelevant emotional face cue was masked whereas in our study the prime was masked (but face cues were clearly visible). Thus, in contrast to our study, the response initiating stimulus was always clearly visible in the Mogg and Bradley study, 1999. In sum, where Mogg and Bradley (1999) showed that a subthreshold emotional cue enhances the visual identification of a subsequent stimulus at the cued location, our study shows that the presentation of an emotional cue enhances the visuomotor processing of subthreshold stimulus *prior* to visual identification.

An emotional boost of covert visuomotor processing could serve to facilitate the quick activation and inhibition of ready action triggers in situations in which there is time-pressure to respond and a more elaborate visual identification of a stimulus is costly. Initially, emotion potentiates the automatic activation of a visuomotor response. However, if the sensory evidence supporting this response is suddenly removed the motor activation is quickly inhibited (Schlaghecken et al., 2006). It has been suggested that the inhibition of a masked prime-induced response may be partly determined by competitive interactions between response-channels where alternating cycles of activation and inhibition continue until one response is selected and all the competing responses are deselected (Praamstra and Seiss, 2005). In this manner, we might speculate that an emotional modulation in visuomotor activation and inhibition may help to facilitate both the rapid selection of correct responses and rapid deselection of erroneous responses during threatening situations.

An interesting question is whether the emotional modulation in visuomotor processing observed in our study is restricted to specific types of emotional stimuli or whether it reflects a more general emotional mechanism. Do our findings extend to other types of visual stimuli (e.g., other emotional facial expressions, affective pictures), and does the pattern of results depend on the specific affective state of the participants? As with most previous studies investigating the influence of emotion on visual processing (Pourtois et al., 2004; Phelps et al., 2006; Bocanegra and Zeelenberg, 2009a,b), we used fearful faces because they have consistently been shown to activate the amygdala (Vuilleumier, 2005). However, it would be interesting to test whether these results extend to other emotional expressions such as anger, happiness, or disgust.

Importantly, the covert visuomotor mechanisms uncovered in the present study constitute a significant conceptual departure from current theorizing in emotion research. Currently, emotional influences on responding are thought to result either from facilitated access or maintenance in visual perception (Yiend, 2010; Bradley et al., 2012). Here, we provide evidence that emotion facilitates visuomotor responding-independent of the perceptual mechanisms that support visual identification. Future accounts of emotional influences in visual RTs may want to incorporate the distinction between covert visuomotor and overt perceptual influences on action.

### Conflict of interest statement

The authors declare that the research was conducted in the absence of any commercial or financial relationships that could be construed as a potential conflict of interest.
